# Potential for pathogenic protein acylation in succinate dehydrogenase-deficient pheochromocytoma and paraganglioma

**DOI:** 10.1530/ERC-26-0243

**Published:** 2026-10-21

**Authors:** Sherry X Zhou, L James Maher

**Affiliations:** ^1^ Department of Biochemistry and Molecular Biology, Mayo Clinic, Rochester, Minnesota, USA; ^2^ Medical Scientist Training Program, Mayo Clinic Graduate School of Biomedical Sciences and Mayo Clinic Alix School of Medicine, Mayo Clinic, Rochester, Minnesota, USA

**Keywords:** succinate dehydrogenase, pheochromocytoma, paraganglioma, protein acylation, acyl-CoA

## Abstract

Historically, the study of cellular metabolism and the cellular proteome have largely focused on specific proteins and metabolites in certain cellular pathways. With the advent of new ‘omics’ technologies, researchers have been able to probe disruptions and alterations in cellular homeostasis on a near proteome-wide and metabolome-wide basis. Yet, the truly interconnected and interdependent nature of the cellular metabolome and proteome has only become more evident with the discovery of proteome-wide protein post-translational modifications (PTMs), such as lysine acylation by acetyl-CoA or succinyl-CoA. Such PTMs link proteome function to fluctuating concentrations of tricarboxylic acid (TCA) cycle metabolites. Tumorigenesis in TCA cycle-defective tumors that upregulate acylating agents, such as succinyl-CoA and acetyl-CoA, provides a relevant context for understanding the breadth of protein acylation in disease. In particular, succinate dehydrogenase (SDH)-loss chromaffin cells can be transformed into the neuroendocrine tumors pheochromocytomas and paragangliomas (PPGLs), but sensitive diagnostic and effective therapeutic options for metastatic disease remain limited. Interrogating cell models of SDH-loss PPGLs provides a unique opportunity to capture the effects of SDH loss on cellular metabolism and protein acylation while exploring new avenues for improved diagnostics and novel therapeutic targets in these rare diseases.

## Pheochromocytoma and paraganglioma

Pheochromocytomas and paragangliomas (PPGLs) are rare tumors arising from chromaffin cells of the adrenal medulla and the paraganglia, respectively. Patients may present with classic symptoms of excess catecholamines, such as episodic hypertension, anxiety or panic or a sense of doom, palpitations, sweating, and tremors, but not all PPGL tumors secrete hormones, nor do all secretory tumors produce large amounts of catecholamines (epinephrine, norepinephrine) (1). Some tumors are biochemically silent, while others may produce dopamine, and these tumors are commonly discovered incidentally on imaging or through symptoms produced by mass effect and compression of surrounding structures (1, 2, 3, 4).

A unique aspect of the PPGL patient population is that up to 30% of patients with PPGLs have hereditary susceptibility, with family members carrying a predisposing germline mutation (5). Individuals who are known to have germline predisposition should have lifelong surveillance (6).

On average, 500–1,600 new PPGL cases are diagnosed annually in the United States (7). Almost one-fifth of PPGLs are metastatic (∼17%) and over 30% of PPGLs are hereditary, often burdening families across multiple generations (8). One-fifth of all PPGLs result from nuclear-encoded mutations in one of four mitochondrial succinate dehydrogenase (SDH) subunits (A–D), with over half of those occurring in SDHB (9) and 10–70% of metastatic PPGLs carrying SDHB mutations (10). Together, PPGL driver mutations have been divided into three main molecular clusters: pseudohypoxia cluster 1 (1A: TCA cycle-related genes; 1B: hypoxia signaling pathway), kinase-signaling cluster 2 (RET, NF1, TMEM127, FGFR1, MAX), and Wnt signaling cluster 3 (MAML3 fusion, somatic CSDE1 gain-of-function) (11, 12). Subsequent acquired mutations in telomere (TERT activation, ATRX inactivation) and chromatin (SETD2) maintenance regulators can alter the likelihood of PPGL recurrence and metastasis.

## TCA cycle defects driving PPGLs

The TCA cycle is a key cellular pathway required for energy production and metabolite catabolism and anabolism, feeding into the electron transport chain (ETC) electron carriers in the form of reduced nicotinamide adenine dinucleotide (NADH) and flavin adenine dinucleotide (FADH_2_) (Fig. 1). The TCA cycle and ETC are also directly connected by the SDH complex, which has a dual role in interconverting succinate and fumarate to yield FAD or FADH_2_ and in shuttling electrons from FADH_2_ into the coenzyme Q pool that feeds into Complex III. Loss of normal TCA cycle enzyme function can lead to accumulation of carboxylate-containing metabolite intermediates, many of which resemble one another in charge, shape, and atomic composition. Thus, an abnormally accumulating metabolite can interfere with enzymatic reactions of similar metabolites through substrate or product inhibition, potentially interfering with normal cellular pathways and leading to tumorigenesis. These ‘oncometabolites’ include the well-known noncanonical TCA cycle metabolite 2-hydroxyglutarate (2-HG), which accumulates as the result of an oncogenic gain-of-function mutation in isocitrate dehydrogenase (IDH) that drives 2-HG production rather than formation of its normal product, 2-oxoglutarate (2-OG, also termed alpha-ketoglutarate/α-KG). It has been well-established that 2-HG accumulation leads to the formation of IDH-mutant gliomas (13), and more recently, high levels of 2-HG have been found to inhibit T-cell function (14). Other known oncometabolites include fumarate and succinate.

**Figure 1:**
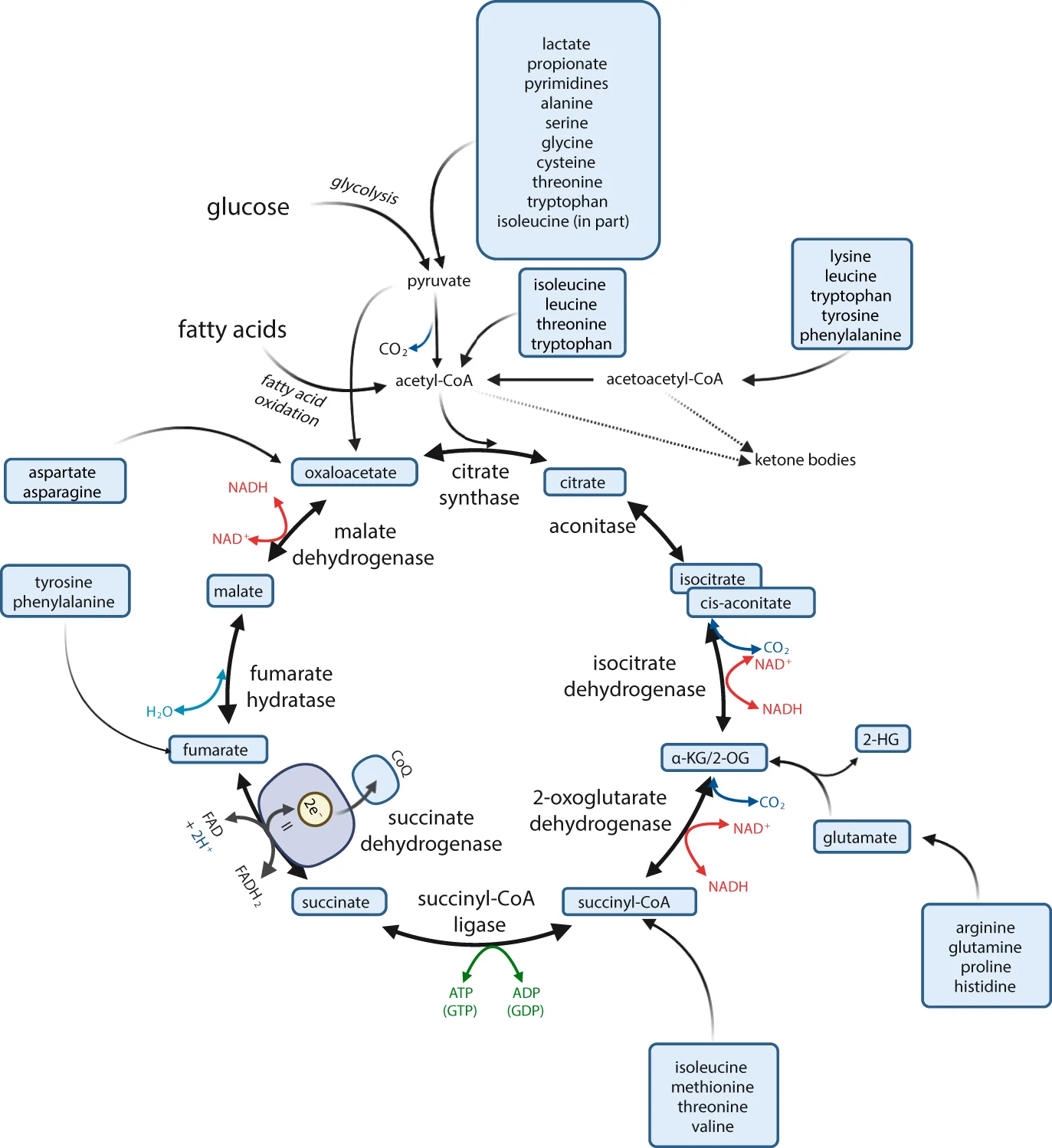
The TCA cycle and its inputs.

Loss of SDH is not unique to PPGL tumors and is also associated with other neuroendocrine neoplasms such as gastrointestinal stromal tumor (GIST) and pituitary adenoma in Carney-Stratakis syndrome, and with renal cell carcinoma (RCC) (15). Loss of fumarate hydratase (FH, also termed fumarase) is also associated with renal cell carcinoma, rarely with PPGL (16) and particularly with hereditary leiomyomatosis and renal cell cancer syndrome (HLRCC) (17).

## SDH loss and metabolism

The details of tumorigenesis (e.g. PPGL, GIST, RCC) upon SDH loss remain unclear. Similar to mutations in IDH, loss of SDH leads to accumulation of an oncometabolite (13, 18). Unlike IDH loss, however, the accumulating oncometabolite, succinate, is a normal participant of the canonical TCA cycle. A leading model for PPGL tumorigenesis focuses on the oncogenic effect of succinate accumulation as the driver for tumor formation (Fig. 2). High succinate concentrations have been shown to competitively inhibit dozens of 2-oxoglutarate-dependent dioxygenases that produce succinate as a byproduct and accommodate succinate as a competitive inhibitor in their active sites (18, 19, 20, 21). These dioxygenases include erasers of histone and DNA methylation such as Jumonji domain (JMJD) histone demethylases and ten-eleven translocation (TET) methylcytosine demethylases (21, 22, 23, 24). Succinate accumulation thus leads to global histone and DNA hypermethylation, the latter of which could block transcription of tumor suppressor genes, resulting in abnormal growth. Other impacted dioxygenases include prolyl hydroxylases required for proper collagen maturation and for tagging HIFs for oxygen-dependent degradation by VHL (18, 19, 21, 24). Through this mechanism, succinate accumulation can induce a ‘pseudohypoxic’ phenotype. This pseudohypoxic state is shared by VHL-loss tumors and SDH-loss tumors, yet SDH-loss tumors are more aggressive (25), suggesting additional tumor-promoting impacts, potentially due to succinate poisoning of other dioxygenases and/or non-succinate-related mechanisms such as formation of excess reactive oxygen species or dysregulation of other reactive metabolites (26, 27, 28, 29, 30). While DNA hypermethylation and pseudohypoxia due to high succinate levels have been identified in SDH-loss cell culture models and in patient tumors, the degree to which these factors drive SDH-loss tumorigenesis remains to be understood.

**Figure 2:**
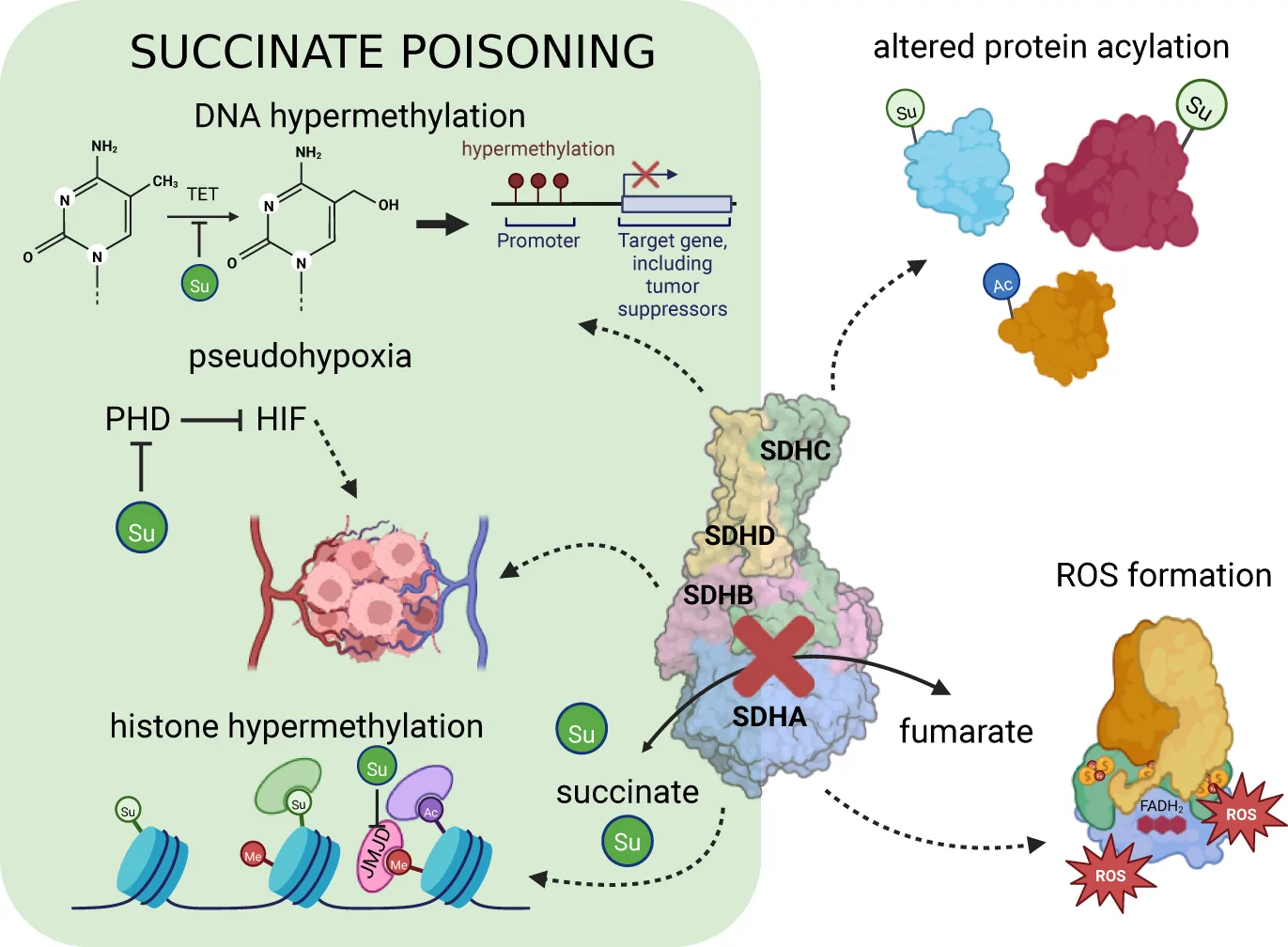
Proposed mechanisms of SDH-loss induced tumorigenesis. Upon SDH loss (red ‘X’), effects due to resulting accumulated succinate (Su) competitive inhibition of dioxygenases is shown within the green box. This includes epigenetic DNA and histone hypermethylation, and inappropriate hypoxia inducible factor (HIF) stabilization. Malformed SDH partial complexes (right, which may or may not assemble) may be sources for damaging and mutagenic reactive oxygen species (ROS; lower right). Finally, the subject of this review, accumulated lysine-reactive acyl-CoA metabolites, may acylate cellular proteins, including enzymes by lysine succinylation or acetylation (shown as bonded Su or Ac at upper right). SDH complex PDB ID: 8GS8 (89).

Importantly, in addition to succinate accumulation, loss of SDH leads to alterations in the levels of other TCA cycle metabolites. In fact, most TCA cycle metabolites appear depleted in both 2D-cultured models of SDH-loss and their SDH-intact counterparts, as well as SDH-loss and SDH-intact patient tumors (31, 32). This dysregulation of the TCA cycle is consistent with the observed highly abnormal mitochondrial morphology (swollen, lacking cristae, containing electron-dense granules) identified in SDH-loss cell models and patient tumors (31, 33, 34, 35), suggesting a significant degree of mitochondrial dysfunction. This apparent mitochondrial dysfunction, and accompanying low energy stress, may also explain the slow-growing character of many PPGL tumors, and the significantly slowed growth rate of SDH-loss cells compared to SDH-intact counterparts in cell culture (31).

Notably, out of the ten direct metabolite intermediates of the TCA cycle, the levels of two particularly labile metabolites, acetyl-CoA and succinyl-CoA, are not routinely quantified and are thus absent from most TCA cycle metabolite panel data. Given that acyl-CoAs are highly reactive with nucleophiles such as protein lysine side chains to form acyllysine residues as post-translational modifications (PTMs), the potential impact of SDH loss on protein acylation merits further characterization. One study has previously shown in mouse fibroblasts that both succinate and succinyl-CoA accumulate upon SDH loss (while other TCA cycle metabolites are depleted). Succinyl-CoA acylates lysine residues of proteins in SDH-loss cells, including histones, thus impacting transcription (36). The importance of post-translational lysine acylation by acyl-CoA accumulation in SDH-loss cells remains to be determined in human PPGLs (37).

## Post-translational lysine acylation

Protein acylation occurs through reactions of acyl-CoAs with the ε-amino group of lysine side chains. Under physiological conditions (pH ∼7.4), the preponderance of lysine side chains are positively charged and nonreactive. However, transient deprotonation of lysine ε-amino groups is constantly occurring, neutralizing ammonium functions to highly active primary amine nucleophiles with electron lone pairs that can react with the electrophilic esters of acyl-CoAs, which have excellent leaving groups. These acyl-CoAs, such as acetyl-CoA, propionyl-CoA, butyryl-CoA, malonyl-CoA, and succinyl-CoA, thus carry activated neutral or negatively charged acyl groups that, upon lysine acylation, alter the charge and size of the lysine side chain (Fig. 3). Acylation occurs both through spontaneous chemical (nonenzymatic) (38, 39) and enzyme-mediated mechanisms that involve multiple acyltransferases discovered over the past decade. These acyltransferases include histone modifiers belonging to the p300, GNAT, and MYST families, as well as KAT2A, OGDH, OCT1, and CPT1A. Fewer deacylases have been characterized, belonging only to two families so far: the histone deacetylase (HDAC) family and the sirtuin family. Most of these enzymes are capable of removing acetylation and possibly other neutral acylation modifications, but only two enzymes have been identified as desuccinylases: sirtuin 5 (SIRT5), a mitochondrial sirtuin, and sirtuin 7 (SIRT7), predominantly found in the nucleus (40, 41, 42).

**Figure 3:**
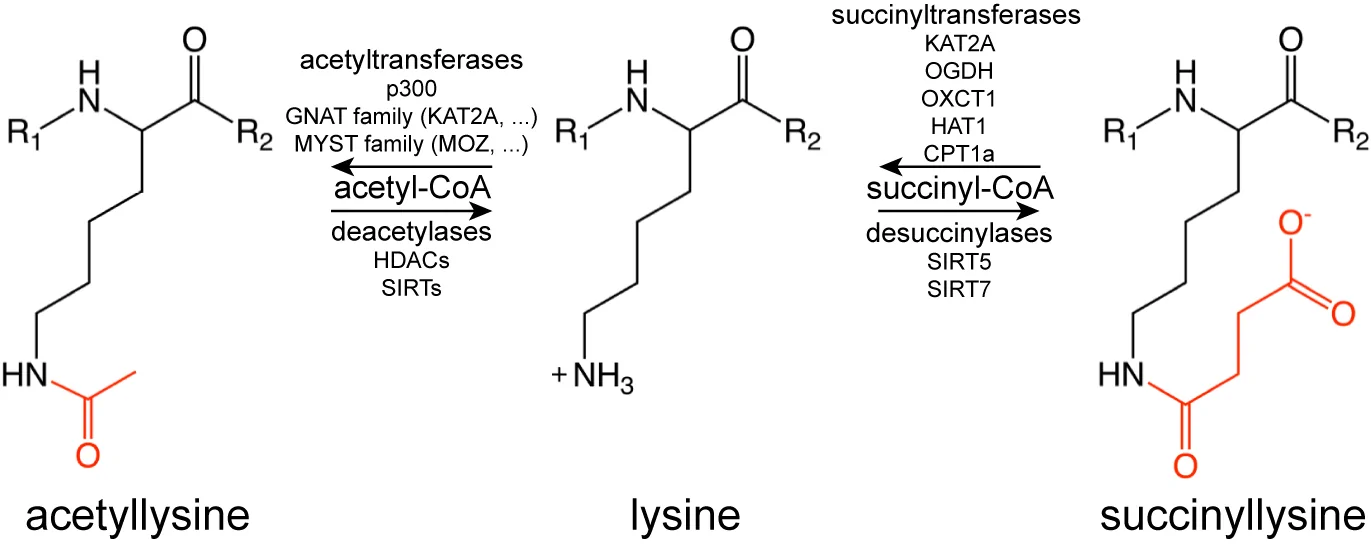
Lysine acetylation and succinylation occur both enzymatically and nonenzymatically, while lysine deacylation is predominantly enzymatic. SDH loss is associated with changes in the levels of many metabolites, including increases in lysine-reactive acetyl-CoA and succinyl-CoA. Both acyl-CoA metabolites can directly acylate the lysine ε-amino side chain, which participates in nucleophilic attack on the acyl-CoA carbonyl with CoA as leaving group. Acylation reactions can be spontaneous or catalyzed by the indicated enzymes. Enzymatic deacylation is catalyzed by other indicated enzymes. In the case of desuccinylation, the reaction mechanism of sirtuin enzymes (e.g. SIRT5, 7) requires NAD^+^, making succinylation stability subject to metabolic changes affecting NAD^+^ concentrations.

Lysine acylation marks, acetylation, and particularly succinylation have been shown to impact protein stability, modifications by additional PTMs, enzymatic activity, and interaction with other macromolecules (43, 44, 45). Histone acetylation is currently the best-studied protein acylation mark and has been shown to increase transcription in numerous studies. More experiments to date have also found that histone succinylation has similar effects in promoting transcription. These acylation impacts on histones likely involve fundamental electrostatic effects, reversing chromatin compaction by reducing favorable histone cation-DNA anion interactions (36, 46).

Protein acylation marks and their regulation by sirtuins have also been implicated in regulation of tumorigenesis and cellular survival (44, 47, 48, 49, 50, 51, 52). However, such studies typically focus on particular proteins or pathways and have not assessed the broader impact of protein acylation homeostasis and the degree of importance of each individual acylation site or collective importance of all acylation sites (or some subset) on a cellular level. Given that most proteins possess critical lysine residues and cells are replete with combinations of more than 20,000 potential proteins, not counting different levels of splicing and post-translationally modified variants, pathological post-translational lysine acylation has yet to be quantified or understood. This gap deserves future attention.

It is noted in passing that certain fumarate hydratase mutations have a rare causative association with PPGLs (16). This is of interest because fumarate accumulation may poison dioxygenases in a manner analogous to succinate. Fumarate can also acylate lysine residues (fumaroylation), and it is unknown if such acylations are pathogenic. Interestingly, fumarate can participate in a distinct and novel post-translational modification of cysteine residues involving Michael addition of the cysteine side chain sulfur at the fumarate carbon–carbon double bond, resulting in essentially irreversible ‘succination’ (named for the appearance of the product). Succination is not an acylation reaction. Its potential pathologic implications are not yet understood (53).

The probability of protein acylation is highly dependent on the compartment(s) through which proteins travel or localize, and the presence or absence of protein acylases and deacylases in those compartments. For example, nonenzymatic acylation is thought to predominantly occur in the mitochondria, where the relatively basic pH of the mitochondrial matrix along with high levels of acyl-CoA substrates, such as acetyl-CoA (imported for the TCA cycle and produced by fatty acid oxidation) and succinyl-CoA (produced in the TCA cycle), increase the probability of chemical acylation (54). In cases of abnormal cellular metabolism, the accumulation of oncometabolites can further distort local acyl-CoA concentrations present to drive pathological acylation (55). The presence of SIRT5 in the mitochondria suggests that cellular regulation of spontaneous chemical acylation occurs, thus implicating dysregulation of mitochondrial acylation as having potentially harmful consequences for cells. Complicating this scenario, sirtuin function requires NAD^+^ as a cofactor (56). NAD^+^ is typically abundant in mitochondria as a product of Complex I of the ETC (regenerating NAD^+^ from NADH). However, under conditions of mitochondrial energy stress, such as SDH loss, sirtuins may not be fueled to reverse enzymatic and nonenzymatic acylation. This pathology could represent a marker of cellular dysfunction, another motivation for further study of pathological protein acylation in SDH-loss cells (37).

Compartmentalization of metabolism and acyl-CoA generation suggest the nucleus and the mitochondria as the major sites of protein acylation in the cell (57, 58, 59). Mitochondrial protein acylation is not as well-studied as nuclear acylation but has been relevant for the maintenance of mitochondrial structure and function (60). In the nucleus, studies have predominantly focused on histone acylation and its effects on nucleosome dynamics and transcription (61, 62). Interestingly, mitochondrial proteins, including TCA cycle enzymes, have been found ‘moonlighting’ in the nucleus, generating acyl-CoAs as substrates for histone acylation (63). Altered histone acylation has also been identified in the context of abnormal metabolism that causes dysregulation of acyl-CoA levels, suggesting that histone acylation reflects the cellular metabolic state (36, 59, 64), integrating metabolism and gene expression. It is unknown if nuclear acyl-CoAs are generated *in situ* or imported from the mitochondria. It should be noted that nuclear histones have also been shown to serve as sinks for excess methyl groups when histone demethylases are inhibited by excess succinate (65).

It is our view that the proper analysis of protein acylation will require both high-throughput, quantitative approaches and targeted biochemical studies to characterize and quantify the landscape of protein acylation and catalog the potential effects of site-specific acylation of particular proteins. Mass spectrometry-based proteomics analysis of samples enriched for a specific acylation mark (e.g. succinyllysine, acetyllysine) enables deeper and more quantitative assessment of alterations of these acylations in samples representing different cellular contexts and pathologies (37). However, as with any antibody-based enrichment method, low-frequency modifications are often missed. Moreover, it is challenging to study multiple acylation types simultaneously in the same sample, particularly when alternative acylations can occur on the same lysine residue. Once particular candidate proteins and their acylation sites are identified, experimental production of the protein with a site-specific acylation remains challenging. Acyl-mimetic mutations have been accepted as approximate analogs for various acylations: glutamate as a mimic for succinyllysine, glutamine for acetyllysine, and arginine for a non-acylable lysine. Other more complex approaches are being developed to exploit click chemistry to attach closer acyllysine analogs as cysteine substitutions (66) or using bacteria engineered to suppress a stop codon with a tRNA carrying an acylated amino acid of interest (e.g. acetyllysine, using a pyrrolysine-charged tRNA from the archaea *Methanosarcina barkeri*) (67). However, not all proteins can be produced successfully in bacterial systems due to differences in chaperones, folding, and post-translational modifications (glycosylation, etc.). More effective methods for studying individual site-specific acylations will be required such that the potential impact of lysine acylation can be understood systematically and rigorously, then broadened to include effects of multiple simultaneous acylations on a single protein. Thus, even with sophisticated mass spectrometry methods, it remains challenging to catalog the range of lysine acylation marks, and how these patterns, and their abundance, change upon SDH loss. The best evidence is currently limited to acetylation and succinylation marks in model systems that possess or lack SDH.

## Protein acylation as a biomarker in disease

Protein acylation is conserved to some extent across all living organisms. The substrates for acylation, acyl-CoAs, exist in both prokaryotic and eukaryotic organisms as a result of various metabolic processes. While protein acylation has predominantly been studied in eukaryotic cells, as the understanding of acylation and deacylation has deepened, so has the interest in understanding the role of acylation in bacteria (68). With the continued characterization of protein acylation in normal and abnormal cells, few studies have attempted to determine how different cell types and tissue types in a single organism may experience different acylation states, let alone across the tissues of the many model organisms employed to further understand human disease. For example, some studies have shown the importance of acylation homeostasis in mouse cardiac and hepatic tissues in the context of metabolism (69, 70, 71), but only when SIRT5 is present or fully knocked out. These studies show that i) mouse tissue protein acylation levels do not necessarily reflect the amount of free acyl-CoA present and ii) proteins from organs and cell lines derived from them do not necessarily share the same acylation patterns. For example, in contrast to the immunoblots of some previous studies (69, 70, 71), but in line with their free acyl-CoA quantifications, we found that cardiac and hepatic tissues display high levels of protein acetylation (Fig. 4) and that cardiac tissue carries a high burden of protein succinylation in murine and porcine samples. Our results clearly show tissue-specific differences in protein acetylation and succinylation, likely due to varying preferences and needs in cellular metabolism. It is unknown why, e.g. normal adrenal tissues of mouse and pig carry a much lower level of lysine acylation than, e.g. heart and lungs. The data in Fig. 4 raise the question of how and why the balance of metabolite substrates and enzyme levels needed for acylation and deacylation differ so greatly even between normal tissues. These results also emphasize the potential for altered protein acylation to have pathological implications.

**Figure 4:**
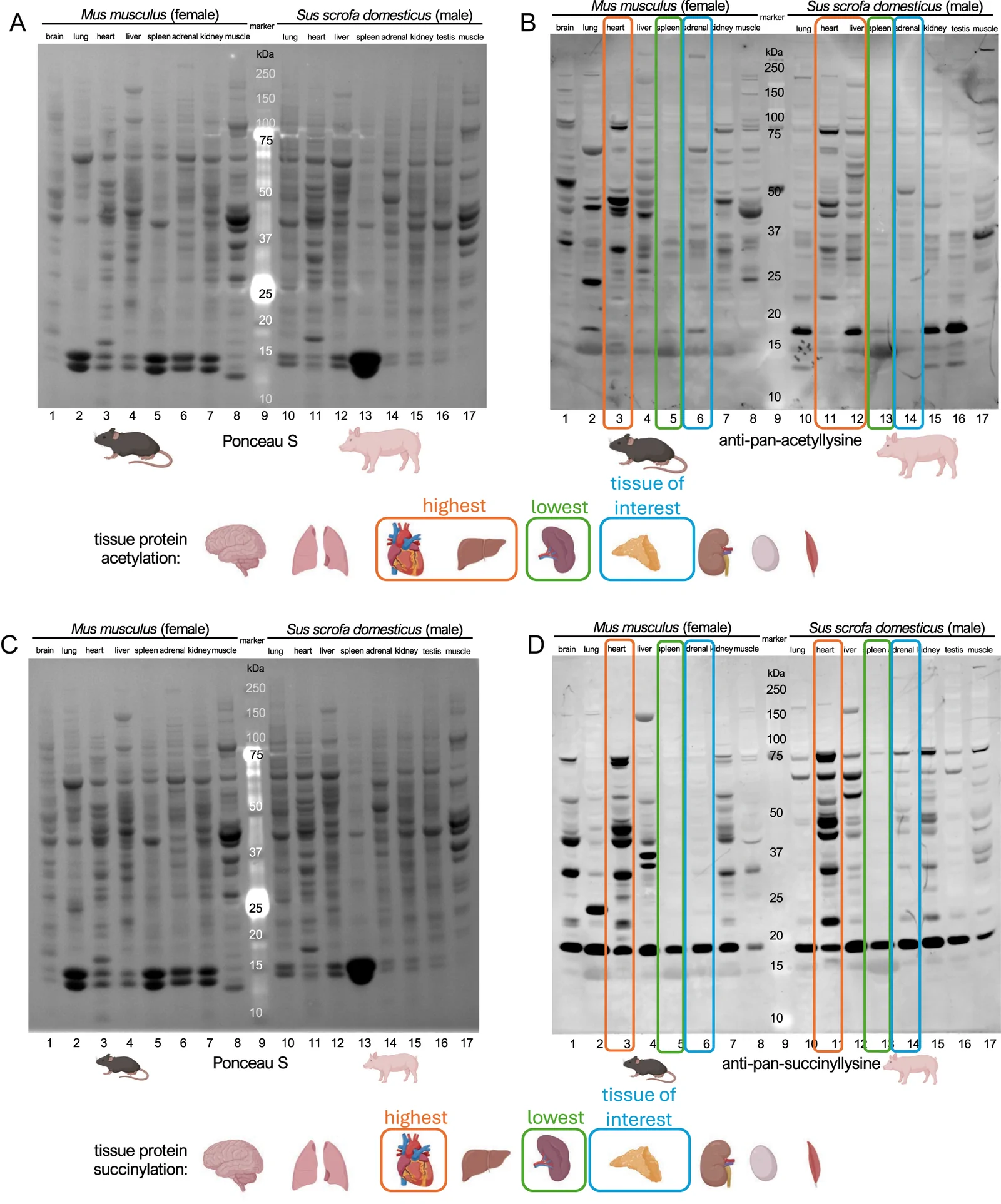
Protein acylation in SDH wild-type mouse and pig tissues. (A and C) Ponceau S staining of membranes in (B and D). (B and D) Immunoblots of tissue lysates from the indicated organs from mouse and pig tissues against protein (B) acetyllysine and (D) succinyllysine residues. Methods: Fresh waste mouse and pig tissues were collected from newly sacrificed animals used for other research studies approved by institutional IACUC. Tissue samples were flash frozen in liquid nitrogen and stored at −80°C before use. Total protein was extracted from 10 mg of each tissue (dissected into millimeter-sized pieces) on ice using RIPA lysis buffer (ChemCruz, USA; sc-24948) with protein deacylation inhibitors (1 μM trichostatin A, (Cayman Chemical, USA; 89730), 20 mM nicotinamide (Millipore Sigma, USA; 72340)) and crushed with a plastic pestle in a microcentrifuge tube. Soluble protein lysate was isolated after clarifying the extract by centrifugation at 15,000 *g*. Quantification of total soluble protein concentration was performed using the BCA protein assay according to the manufacturer’s protocol (Pierce, USA; 23225). Protein lysates were denatured and reduced (Invitrogen, USA; NP0004) in LDS (Invitrogen, USA; NP0007) at 95°C for 5 min immediately after protein quantification. 40 μg of lysate was loaded per lane on 4–12% Bis-Tris denaturing polyacrylamide gels (Invitrogen, USA) and subjected to SDS-PAGE in reducing MES buffer (Invitrogen, USA; NP0002, NP0005). Proteins were then transferred to a PVDF membrane (Bio-Rad, USA; 1620174) in transfer buffer (Invitrogen, USA; NP00061) containing 20% methanol. Membranes were then briefly stained with Ponceau S, imaged on a Typhoon 5 biomolecular imager (Amersham, UK), destained with deionized water, and blocked in 5% non-fat dairy milk dissolved in TBST for 2 h at room temperature. The membrane was washed with TBST before incubating with primary antibodies (anti-acetyllysine (PTM BIO, USA; rabbit monoclonal PTM-105RM, 1:500)), or anti-succinyllysine (PTM BIO, China; rabbit polyclonal PTM-401, 1:500) diluted into TBST overnight with gentle rocking at 4°C overnight. The membrane was then washed with TBST prior to incubation with fluorescent secondary antibodies (LI-COR, USA; IRDye 680RD goat anti-rabbit 926-68071, 1:20,000 and IRDye 800CW goat anti-mouse 926-32210, 1:20,000) with gentle rocking for 15–20 min at room temperature. The membrane was washed with TBST, then with TBS prior to imaging on a Typhoon 5 fluorimager.

Because the substrates for protein acylation arise from key energy transduction pathways, these PTMs link metabolic homeostasis and acylation homeostasis. Many studies have highlighted the unique roles of acetyl-CoA and succinyl-CoA as both metabolic intermediates, chemical messengers, and protein modifiers, with their fluctuating concentrations serving to integrate the metabolome, the proteome, and the transcriptome (72, 73, 74, 75, 76, 77). Further connections have been made on the tissue and mitochondrial levels, focusing on how whole-body metabolism and diet can impact protein acylation homeostasis (78, 79). On the cellular level, links have been noted between cell growth and metabolic state and acylation homeostasis (80).

Some disease states have direct impacts on concentrations of acylation substrates and are thus expected to directly impact protein acylation. One rare human disease involves loss of succinyl-CoA synthetase (encoded by *SUCLG2*), the TCA cycle enzyme complex responsible for interconverting succinyl-CoA and succinate, producing adenosine triphosphate (ATP) or guanosine triphosphate (GTP) in the ‘forward’ direction when producing succinate, or burning ATP or GTP in the ‘reverse’ direction. Mutations in subunits of succinyl-CoA synthetase have been shown to cause accumulation of succinyl-CoA in patient cells as well as accumulation of protein succinylation (81). Furthermore, global protein hypersuccinylation in the setting of succinyl-CoA synthetase loss is associated with decreased survival and impaired cellular function in zebrafish and mouse models of this disease (81, 82). Interestingly, *SUCLG2* can also be mutated in PPGLs (83).

Altered protein acylation has also been implicated in abnormal cellular proliferation, mitochondrial dysfunction, and subsequent resistance to apoptosis unique to some cell types, while other cell types appear more resistant to the effects of altered protein acylation homeostasis (84, 85, 86). Other studies have linked abnormal protein acylation homeostasis to neurodegenerative disease and cancer through the shared dysregulation of mitochondrial metabolism (87, 88). The dysregulation of acyl-CoA levels that occurs in the setting of SDH loss may thus also play a unique role in the tumorigenesis of SDH-loss PPGLs and contribute to the increased aggressiveness of SDH-loss tumors (37). Quantifiable differences in protein acylation may be a shared feature of SDH-loss tumors, as well as other diseases with altered mitochondrial metabolism, and could potentially serve as a better marker of abnormal metabolism and disease. This possibility merits future detailed examination.

We hypothesize that changes in levels of acylation across cells and in different subcellular compartments, as well as altered positions of acylation marks on sensitive proteins (including chromatin proteins involved in epigenetic control), have the potential to contribute to PPGL tumorigenesis and to serve as biomarkers.

## Declaration of interest

The authors declare that there is no conflict of interest that could be perceived as prejudicing the impartiality of this work.

## Funding

This work was funded by generous grants from the Paradifference Foundationhttps://doi.org/10.13039/100016778 to LJM. This work was also supported by the Mayo Clinic Graduate School of Biomedical Sciences (SXZ) and Mayo Clinic Medical Scientist Training Program NIH institutional training grant T32 GM145408 (HHS | NIH | National Institute of General Medical Scienceshttps://doi.org/10.13039/100000057) (SXZ).

## Author contribution statement

SXZ and LJM wrote the manuscript. All authors read and approved the final manuscript.

## References

[1] Lenders JWM, Kerstens MN, Amar L, et al. Genetics, diagnosis, management and future directions of research of phaeochromocytoma and paraganglioma: a position statement and consensus of the Working Group on Endocrine Hypertension of the European Society of Hypertension. J Hypertens 2020 38 1443–1456. (10.1097/hjh.0000000000002438) PMC7486815 32412940

[2] Geroula A, Deutschbein T, Langton K, et al. Pheochromocytoma and paraganglioma: clinical feature-based disease probability in relation to catecholamine biochemistry and reason for disease suspicion. Eur J Endocrinol 2019 181 409–420. (10.1530/eje-19-0159) 31370000

[3] Fassnacht M, Arlt W, Bancos I, et al. Management of adrenal incidentalomas: European Society of Endocrinology Clinical Practice Guideline in collaboration with the European Network for the Study of Adrenal tumors. Eur J Endocrinol 2016 175 G1–G34. (10.1530/eje-16-0467) 27390021

[4] Brain KL, Kay J & Shine B. Measurement of urinary metanephrines to screen for pheochromocytoma in an unselected hospital referral population. Clin Chem 2006 52 2060–2064. (10.1373/clinchem.2006.070805) PMC2640466 16990424

[5] Fishbein L, Merrill S, Fraker DL, et al. Inherited mutations in pheochromocytoma and paraganglioma: why all patients should be offered genetic testing. Ann Surg Oncol 2013 20 1444–1450. (10.1245/s10434-013-2942-5) PMC4291281 23512077

[6] Nolting S, Bechmann N, Taieb D, et al. Personalized management of pheochromocytoma and paraganglioma. Endocr Rev 2022 43 199–239. (10.1210/endrev/bnab019) PMC8905338 34147030

[7] Beard AM, Sheps SG, Kurland LT, et al. Occurrence of pheochromocytoma in Rochester, Minnesota, 1950 through 1979. Mayo Clin Proc 1983 58 802–804. (10.1016/s0025-6196(25)18873-3) 6645626

[8] Jimenez C, Rohren E, Habra MA, et al. Current and future treatments for malignant pheochromocytoma and sympathetic paraganglioma. Curr Oncol Rep 2013 15 356–371. (10.1007/s11912-013-0320-x) 23674235

[9] Favier J, Amar L & Gimenez-Roqueplo AP. Paraganglioma and phaeochromocytoma: from genetics to personalized medicine. Nat Rev Endocrinol 2015 11 101–111. (10.1038/nrendo.2014.188) 25385035

[10] Pasini B & Stratakis CA. SDH mutations in tumorigenesis and inherited endocrine tumours: lesson from the phaeochromocytoma-paraganglioma syndromes. J Intern Med 2009 266 19–42. (10.1111/j.1365-2796.2009.02111.x) PMC3163304 19522823

[11] Fishbein L, Leshchiner I, Walter V, et al. Comprehensive molecular characterization of pheochromocytoma and paraganglioma. Cancer Cell 2017 31 181–193. (10.1016/j.ccell.2017.01.001) PMC5643159 28162975

[12] Crona J, Taieb D & Pacak K. New perspectives on pheochromocytoma and paraganglioma: toward a molecular classification. Endocr Rev 2017 38 489–515. (10.1210/er.2017-00062) PMC5716829 28938417

[13] Dang L, White DW, Gross S, et al. Cancer-associated IDH1 mutations produce 2-hydroxyglutarate. Nature 2009 462 739–744. (10.1038/nature08617) PMC2818760 19935646

[14] Notarangelo G, Spinelli JB, Perez EM, et al. Oncometabolite d-2HG alters T cell metabolism to impair CD8(+) T cell function. Science 2022 377 1519–1529. (10.1126/science.abj5104) PMC9629749 36173860

[15] Bardella C, Pollard PJ & Tomlinson I. SDH mutations in cancer. Biochim Biophys Acta 2011 1807 1432–1443. (10.1016/j.bbabio.2011.07.003) 21771581

[16] Zavoshi S, Lu E, Boutros PC, et al. Fumarate hydratase variants and their association with paraganglioma/pheochromocytoma. Urology 2023 176 106–114. (10.1016/j.urology.2022.11.053) 36773955

[17] Orrego JJ & Chorny JA. Hereditary leiomyomatosis and renal cell cancer (HLRCC), pheochromocytoma (PCC)/paraganglioma (PGL) and germline fumarate hydratase (FH) variants. Endocrinol Diabetes Metab Case Rep 2024 2024 e240073. (10.1530/EDM-24-0073) PMC11737469 39705504

[18] Selak MA, Armour SM, MacKenzie ED, et al. Succinate links TCA cycle dysfunction to oncogenesis by inhibiting HIF-alpha prolyl hydroxylase. Cancer Cell 2005 7 77–85. (10.1016/j.ccr.2004.11.022) 15652751

[19] Pollard PJ, Briere JJ, Alam NA, et al. Accumulation of Krebs cycle intermediates and over-expression of HIF1alpha in tumours which result from germline FH and SDH mutations. Hum Mol Genet 2005 14 2231–2239. (10.1093/hmg/ddi227) 15987702

[20] Selak MA, Duran RV & Gottlieb E. Redox stress is not essential for the pseudo-hypoxic phenotype of succinate dehydrogenase deficient cells. Biochim Biophys Acta 2006 1757 567–572. (10.1016/j.bbabio.2006.05.015) 16797480

[21] Morin A, Goncalves J, Moog S, et al. TET-mediated hypermethylation primes SDH-deficient cells for HIF2alpha-driven mesenchymal transition. Cell Rep 2020 30 4551–4566.e7. (10.1016/j.celrep.2020.03.022) 32234487

[22] Smith EH, Janknecht R & Maher LJ 3rd. Succinate inhibition of alpha-ketoglutarate-dependent enzymes in a yeast model of paraganglioma. Hum Mol Genet 2007 16 3136–3148. (10.1093/hmg/ddm275) 17884808

[23] Cervera AM, Bayley JP, Devilee P, et al. Inhibition of succinate dehydrogenase dysregulates histone modification in mammalian cells. Mol Cancer 2009 8 89. (10.1186/1476-4598-8-89) PMC2770992 19849834

[24] Xiao M, Yang H, Xu W, et al. Inhibition of alpha-KG-dependent histone and DNA demethylases by fumarate and succinate that are accumulated in mutations of FH and SDH tumor suppressors. Genes Dev 2012 26 1326–1338. (10.1101/gad.191056.112) PMC3387660 22677546

[25] Dahia PLM, Clifton-Bligh R, Gimenez-Roqueplo AP, et al. Hereditary endorine tumours: current state-of-the-art and research opportunities: metastatic pheochromocytomas and paragangliomas: proceedings of the MEN2019 workshop. Endocr Relat Cancer 2020 27 T41–T52. (10.1530/erc-19-0435) PMC7334096 32069214

[26] Slane BG, Aykin-Burns N, Smith BJ, et al. Mutation of succinate dehydrogenase subunit C results in increased O_2_.-oxidative stress, and genomic instability. Cancer Res 2006 66 7615–7620. (10.1158/0008-5472.can-06-0833) 16885361

[27] Szeto SSW, Reinke SN, Sykes BD, et al. Ubiquinone-binding site mutations in the *Saccharomyces cerevisiae* succinate dehydrogenase generate superoxide and lead to the accumulation of succinate. J Biol Chem 2007 282 27518–27526. (10.1074/jbc.m700601200) 17636259

[28] Drose S. Differential effects of complex II on mitochondrial ROS production and their relation to cardioprotective pre- and postconditioning. Biochim Biophys Acta 2013 1827 578–587. (10.1016/j.bbabio.2013.01.004) 23333272

[29] Wagner GR & Hirschey MD. Nonenzymatic protein acylation as a carbon stress regulated by sirtuin deacylases. Mol Cell 2014 54 5–16. (10.1016/j.molcel.2014.03.027) PMC4040445 24725594

[30] Senoo-Matsuda N, Hartman PS, Akatsuka A, et al. A complex II defect affects mitochondrial structure, leading to ced-3- and ced-4-dependent apoptosis and aging. J Biol Chem 2003 278 22031–22036. (10.1074/jbc.m211377200) 12672828

[31] Al Khazal FJ, Bhat SM, Zhu Y, et al. Similar deficiencies, different outcomes: succinate dehydrogenase loss in adrenal medulla vs. fibroblast cell culture models of paraganglioma. Cancer Metab 2024 12 39. (10.1186/s40170-024-00369-9) PMC11668036 39716277

[32] Lussey-Lepoutre C, Hollinshead KE, Ludwig C, et al. Loss of succinate dehydrogenase activity results in dependency on pyruvate carboxylation for cellular anabolism. Nat Commun 2015 6 8784. (10.1038/ncomms9784) PMC4632646 26522426

[33] D'Antongiovanni V, Martinelli S, Richter S, et al. The microenvironment induces collective migration in SDHB-silenced mouse pheochromocytoma spheroids. Endocr Relat Cancer 2017 24 555–564. (10.1530/erc-17-0212) PMC7441822 28830936

[34] Douwes Dekker PB, Hogendoorn PC, Kuipers-Dijkshoorn N, et al. SDHD mutations in head and neck paragangliomas result in destabilization of complex II in the mitochondrial respiratory chain with loss of enzymatic activity and abnormal mitochondrial morphology. J Pathol 2003 201 480–486. (10.1002/path.1461) 14595761

[35] Szarek E, Ball ER, Imperiale A, et al. Carney triad, SDH-deficient tumors, and Sdhb+/- mice share abnormal mitochondria. Endocr Relat Cancer 2015 22 345–352. (10.1530/erc-15-0069) PMC4433412 25808178

[36] Smestad J, Erber L, Chen Y, et al. Chromatin succinylation correlates with active gene expression and is perturbed by defective TCA cycle metabolism. iScience 2018 2 63–75. (10.1016/j.isci.2018.03.012) PMC5993049 29888767

[37] Zhou SX, Madden B, Charlesworth MC, et al. Distinct proteomic and acylproteomic adaptations to succinate dehydrogenase loss in two cell contexts. bioRxiv 2025 2025.08.01.668168. (10.1101/2025.08.01.668168)

[38] Wagner GR, Bhatt DP, O'Connell TM, et al. A class of reactive acyl-CoA species reveals the non-enzymatic origins of protein acylation. Cell Metab 2017 25 823–837.e8. (10.1016/j.cmet.2017.03.006) PMC5399522 28380375

[39] Baeza J, Smallegan MJ & Denu JM. Site-specific reactivity of nonenzymatic lysine acetylation. ACS Chem Biol 2015 10 122–128. (10.1021/cb500848p) PMC4301072 25555129

[40] Du J, Zhou Y, Su X, et al. Sirt5 is a NAD-dependent protein lysine demalonylase and desuccinylase. Science 2011 334 806–809. (10.1126/science.1207861) PMC3217313 22076378

[41] Park J, Chen Y, Tishkoff DX, et al. SIRT5-mediated lysine desuccinylation impacts diverse metabolic pathways. Mol Cell 2013 50 919–930. (10.1016/j.molcel.2013.06.001) PMC3769971 23806337

[42] Wang M & Lin H. Understanding the function of mammalian sirtuins and protein lysine acylation. Annu Rev Biochem 2021 90 245–285. (10.1146/annurev-biochem-082520-125411) 33848425

[43] Lee JM, Hammaren HM, Savitski MM, et al. Control of protein stability by post-translational modifications. Nat Commun 2023 14 201. (10.1038/s41467-023-35795-8) PMC9839724 36639369

[44] Andersen JL, Thompson JW, Lindblom KR, et al. A biotin switch-based proteomics approach identifies 14-3-3zeta as a target of Sirt1 in the metabolic regulation of caspase-2. Mol Cell 2011 43 834–842. (10.1016/j.molcel.2011.07.028) PMC3417139 21884983

[45] Inuzuka H, Gao D, Finley LW, et al. Acetylation-dependent regulation of Skp2 function. Cell 2012 150 179–193. (10.1016/j.cell.2012.05.038) PMC3595190 22770219

[46] Pineiro M, Hernandez F & Palacian E. Succinylation of histone amino groups facilitates transcription of nucleosomal cores. Biochim Biophys Acta 1992 1129 183–187. (10.1016/0167-4781(92)90485-i) 1730057

[47] Sundaresan NR, Pillai VB, Wolfgeher D, et al. The deacetylase SIRT1 promotes membrane localization and activation of Akt and PDK1 during tumorigenesis and cardiac hypertrophy. Sci Signal 2011 4 ra46. (10.1126/scisignal.2001465) 21775285

[48] Luo J, Nikolaev AY, Imai S, et al. Negative control of p53 by Sir2alpha promotes cell survival under stress. Cell 2001 107 137–148. (10.1016/s0092-8674(01)00524-4) 11672522

[49] Vaziri H, Dessain SK, Eaton EN, et al. hSIR2(SIRT1) functions as an NAD-dependent p53 deacetylase. Cell 2001 107 149–159. (10.1016/s0092-8674(01)00527-x) 11672523

[50] Lin R, Tao R, Gao X, et al. Acetylation stabilizes ATP-citrate lyase to promote lipid biosynthesis and tumor growth. Mol Cell 2013 51 506–518. (10.1016/j.molcel.2013.07.002) PMC4180208 23932781

[51] Seo KS, Park JH, Heo JY, et al. SIRT2 regulates tumour hypoxia response by promoting HIF-1alpha hydroxylation. Oncogene 2015 34 1354–1362. (10.1038/onc.2014.76) 24681946

[52] Guarani V, Deflorian G, Franco CA, et al. Acetylation-dependent regulation of endothelial Notch signalling by the SIRT1 deacetylase. Nature 2011 473 234–238. (10.1038/nature09917) PMC4598045 21499261

[53] Guberovic I & Frezza C. Functional implications of fumarate-induced cysteine succination. Trends Biochem Sci 2024 49 775–790. (10.1016/j.tibs.2024.05.003) 38876954

[54] Wagner GR & Payne RM. Widespread and enzyme-independent N-epsilon-acetylation and N-epsilon-succinylation of proteins in the chemical conditions of the mitochondrial matrix. J Biol Chem 2013 288 29036–29045. (10.1074/jbc.m113.486753) PMC3790002 23946487

[55] Fu Y, Yu J, Li F, et al. Oncometabolites drive tumorigenesis by enhancing protein acylation: from chromosomal remodelling to nonhistone modification. J Exp Clin Cancer Res 2022 41 144. (10.1186/s13046-022-02338-w) PMC9013066 35428309

[56] Anderson KA, Madsen AS, Olsen CA, et al. Metabolic control by sirtuins and other enzymes that sense NAD(+), NADH, or their ratio. Biochim Biophys Acta Bioenerg 2017 1858 991–998. (10.1016/j.bbabio.2017.09.005) PMC5648639 28947253

[57] Trefely S, Lovell CD, Snyder NW, et al. Compartmentalised acyl-CoA metabolism and roles in chromatin regulation. Mol Metab 2020 38 100941. (10.1016/j.molmet.2020.01.005) PMC7300382 32199817

[58] Sivanand S, Viney I & Wellen KE. Spatiotemporal control of Acetyl-CoA metabolism in chromatin regulation. Trends Biochem Sci 2018 43 61–74. (10.1016/j.tibs.2017.11.004) PMC5741483 29174173

[59] Nitsch S, Zorro Shahidian L & Schneider R. Histone acylations and chromatin dynamics: concepts, challenges, and links to metabolism. EMBO Rep 2021 22 e52774. (10.15252/embr.202152774) PMC8406397 34159701

[60] Guhathakurta S, Erdogdu NU, Hoffmann JJ, et al. COX17 acetylation via MOF-KANSL complex promotes mitochondrial integrity and function. Nat Metab 2023 5 1931–1952. (10.1038/s42255-023-00904-w) PMC10663164 37813994

[61] Zorro Shahidian L, Haas M, Le Gras S, et al. Succinylation of H3K122 destabilizes nucleosomes and enhances transcription. EMBO Rep 2021 22 e51009. (10.15252/embr.202051009) PMC7926236 33512761

[62] Liu J, Shangguan Y, Tang D, et al. Histone succinylation and its function on the nucleosome. J Cell Mol Med 2021 25 7101–7109. (10.1111/jcmm.16676) PMC8335665 34160884

[63] Li W, Long Q, Wu H, et al. Nuclear localization of mitochondrial TCA cycle enzymes modulates pluripotency via histone acetylation. Nat Commun 2022 13 7414. (10.1038/s41467-022-35199-0) PMC9718843 36460681

[64] Simithy J, Sidoli S, Yuan ZF, et al. Characterization of histone acylations links chromatin modifications with metabolism. Nat Commun 2017 8 1141. (10.1038/s41467-017-01384-9) PMC5656686 29070843

[65] Perez MF & Sarkies P. Histone methyltransferase activity affects metabolism in human cells independently of transcriptional regulation. PLoS Biol 2023 21 e3002354. (10.1371/journal.pbio.3002354) PMC10602318 37883365

[66] Afonso CF, Marques MC, Antonio JPM, et al. Cysteine-assisted click-chemistry for proximity-driven, site-specific acetylation of histones. Angew Chem Int Ed Engl 2022 134 e202208543. (10.1002/ange.202208543) PMC9828500 36124857

[67] Neumann H, Peak-Chew SY & Chin JW. Genetically encoding N(epsilon)-acetyllysine in recombinant proteins. Nat Chem Biol 2008 4 232–234. (10.1038/nchembio.73) 18278036

[68] VanDrisse CM & Escalante-Semerena JC. Protein acetylation in bacteria. Annu Rev Microbiol 2019 73 111–132. (10.1146/annurev-micro-020518-115526) PMC6736716 31091420

[69] Yu J, Sadhukhan S, Noriega LG, et al. Metabolic characterization of a Sirt5 deficient mouse model. Sci Rep 2013 3 2806. (10.1038/srep02806) PMC3786297 24076663

[70] Sadhukhan S, Liu X, Ryu D, et al. Metabolomics-assisted proteomics identifies succinylation and SIRT5 as important regulators of cardiac function. Proc Natl Acad Sci U S A 2016 113 4320–4325. (10.1073/pnas.1519858113) PMC4843474 27051063

[71] Rardin MJ, He W, Nishida Y, et al. SIRT5 regulates the mitochondrial lysine succinylome and metabolic networks. Cell Metab 2013 18 920–933. (10.1016/j.cmet.2013.11.013) PMC4105152 24315375

[72] Yang Y & Gibson GE. Succinylation links metabolism to protein functions. Neurochem Res 2019 44 2346–2359. (10.1007/s11064-019-02780-x) PMC6755074 30903449

[73] Choudhary C, Weinert BT, Nishida Y, et al. The growing landscape of lysine acetylation links metabolism and cell signalling. Nat Rev Mol Cell Biol 2014 15 536–550. (10.1038/nrm3841) 25053359

[74] Shi L & Tu BP. Acetyl-CoA and the regulation of metabolism: mechanisms and consequences. Curr Opin Cell Biol 2015 33 125–131. (10.1016/j.ceb.2015.02.003) PMC4380630 25703630

[75] Pietrocola F, Galluzzi L, Bravo-San Pedro JM, et al. Acetyl coenzyme A: a central metabolite and second messenger. Cell Metab 2015 21 805–821. (10.1016/j.cmet.2015.05.014) 26039447

[76] Lukey MJ, Greene KS & Cerione RA. Lysine succinylation and SIRT5 couple nutritional status to glutamine catabolism. Mol Cell Oncol 2020 7 1735284. (10.1080/23723556.2020.1735284) PMC7199738 32391426

[77] Xu H, Chen X, Xu X, et al. Lysine acetylation and succinylation in HeLa cells and their essential roles in response to UV-induced stress. Sci Rep 2016 6 30212. (10.1038/srep30212) PMC4959001 27452117

[78] Meyer JG, Softic S, Basisty N, et al. Temporal dynamics of liver mitochondrial protein acetylation and succinylation and metabolites due to high fat diet and/or excess glucose or fructose. PLoS One 2018 13 e0208973. (10.1371/journal.pone.0208973) PMC6306174 30586434

[79] Liu X, Sadhukhan S, Sun S, et al. High-resolution metabolomics with Acyl-CoA profiling reveals widespread remodeling in response to diet. Mol Cell Proteomics 2015 14 1489–1500. (10.1074/mcp.m114.044859) PMC4458715 25795660

[80] Yuan T, Keijer J, Guo AH, et al. An optimized desuccinylase activity assay reveals a difference in desuccinylation activity between proliferative and differentiated cells. Sci Rep 2020 10 17030. (10.1038/s41598-020-72833-7) PMC7552388 33046741

[81] Gut P, Matilainen S, Meyer JG, et al. SUCLA2 mutations cause global protein succinylation contributing to the pathomechanism of a hereditary mitochondrial disease. Nat Commun 2020 11 5927. (10.1038/s41467-020-19743-4) PMC7684291 33230181

[82] Lancaster MS, Kim B, Doud EH, et al. Loss of succinyl-CoA synthetase in mouse forebrain results in hypersuccinylation with perturbed neuronal transcription and metabolism. Cell Rep 2023 42 113241. (10.1016/j.celrep.2023.113241) PMC10683835 37819759

[83] Hadrava Vanova K, Pang Y, Krobova L, et al. Germline SUCLG2 variants in patients with pheochromocytoma and paraganglioma. J Natl Cancer Inst 2022 114 130–138. (10.1093/jnci/djab158) PMC8755484 34415331

[84] Yang X, Wang Z, Li X, et al. SHMT2 desuccinylation by SIRT5 drives cancer cell proliferation. Cancer Res 2018 78 372–386. (10.1158/0008-5472.can-17-1912) 29180469

[85] Lagerwaard B, Pougovkina O, Bekebrede AF, et al. Increased protein propionylation contributes to mitochondrial dysfunction in liver cells and fibroblasts, but not in myotubes. J Inherit Metab Dis 2021 44 438–449. (10.1002/jimd.12296) PMC8049071 32740932

[86] Li F, He X, Ye D, et al. NADP(+)-IDH mutations promote hypersuccinylation that impairs mitochondria respiration and induces apoptosis resistance. Mol Cell 2015 60 661–675. (10.1016/j.molcel.2015.10.017) 26585387

[87] Yang Y, Tapias V, Acosta D, et al. Altered succinylation of mitochondrial proteins, APP and tau in Alzheimer’s disease. Nat Commun 2022 13 159. (10.1038/s41467-021-27572-2) PMC8748865 35013160

[88] Guertin DA & Wellen KE. Acetyl-CoA metabolism in cancer. Nat Rev Cancer 2023 23 156–172. (10.1038/s41568-022-00543-5) PMC11137663 36658431

[89] Du Z, Zhou X, Lai Y, et al. Structure of the human respiratory complex II. Proc Natl Acad Sci U S A 2023 120 e2216713120. (10.1073/pnas.2216713120) PMC10161127 37098072

